# Process Design and Economics of On-Site Cellulase Production on Various Carbon Sources in a Softwood-Based Ethanol Plant

**DOI:** 10.4061/2010/734182

**Published:** 2010-06-28

**Authors:** Zsolt Barta, Krisztina Kovacs, Kati Reczey, Guido Zacchi

**Affiliations:** ^1^Department of Applied Biotechnology and Food Science, Budapest University of Technology and Economics, Szt. Gellért tér 4, 1111 Budapest, Hungary; ^2^Department of Chemical Engineering, Lund University, P.O. Box 124, 221 00 Lund, Sweden

## Abstract

On-site cellulase enzyme fermentation in a softwood-to-ethanol process, based on SO_2_-catalysed steam pretreatment followed by simultaneous saccharification and fermentation, was investigated from a techno-economic aspect using Aspen Plus*©* and Aspen Icarus Process Evaluator*©* softwares. The effect of varying the carbon source of enzyme fermentation, at constant protein and mycelium yields, was monitored through the whole process. Enzyme production step decreased the overall ethanol yield (270 L/dry tonne of raw material in the case of purchased enzymes) by 5–16 L/tonne. Capital cost was found to be the main cost contributor to enzyme fermentation, constituting to 60–78% of the enzyme production cost, which was in the range of 0.42–0.53 SEK/L ethanol. The lowest minimum ethanol selling prices (4.71 and 4.82 SEK/L) were obtained in those scenarios, where pretreated liquid fraction supplemented with molasses was used as carbon source. In some scenarios, on-site enzyme fermentation was found to be a feasible alternative.

## 1. Introduction

Production of ethanol from lignocellulosic materials is a very complex process which consists of various interdependent steps, such as pretreatment of the raw material, enzymatic hydrolysis of the polysaccharides into sugar monomers, fermentation of the sugars to ethanol, and purification of ethanol. Since the process has not yet been demonstrated on a commercial scale, only a limited number of studies are available on its techno-economic aspects, and large variations in the estimated overall ethanol production costs (from about 0.93 to 5.49 SEK/L ethanol) can be seen, due to differences in the process design and in the assumptions used in the studies [1–10]. One of the most important parameters that influences the production costs is the annual capacity of the plants, that is, lower production costs are usually obtained for plants that process above 650 000 tons of dry raw material per year. Other differences are found in the conversion technologies, the types of raw materials used, the overall ethanol yields assumed, the investment parameters, and whether utilities such as process steam and electricity are included as costs in the assessment [3].

According to recent techno-economic evaluations, the main contributors to the overall costs of producing ethanol from biomass are the raw material (30%–40%) and the capital investment (30%–45%), followed by the cellulase enzymes (10%–20%) [6, 7, 11–13]. The cost of cellulases not only represents a significant part in the overall production costs but is also one of the most uncertain parameters in the evaluations [3]. Most authors assume that cellulases are purchased from enzyme manufacturers and calculate with an estimated future enzyme price, which varies from about 0.2 to 0.7 SEK/L ethanol in the investigations reviewed in [1, 6, 7, 11–15]. However, some other studies presume that on-site or near-site production on cheap lignocellulosic raw materials will be desirable to meet the targeted enzyme costs of <0.5 SEK/L [8, 16–20]. In any case, improvement of cellulolytic microorganisms, enhancement of the hydrolytic capacity of cellulases, and optimization of the technology of enzyme production are essential today in order to further reduce the enzyme costs for the biomass-to-bioethanol process.

Spruce is the most abundant wood in Sweden, and it was shown to be a suitable raw material for bioethanol production in several studies [21–24]. Hypercellulolytic mutants of *Trichoderma reesei*, the most widely used fungus for cellulase production, were reported to grow well, and secrete high amounts of cellulolytic enzymes on steam-pretreated spruce [25, 26]. The most economical way of employing the enzymes produced would be the direct use of whole crude fermentation broths, containing fungal cells and substrate residues, in order to avoid expensive cell removal, enzyme concentration, and purification steps. Previous investigations showed that due to the effect of mycelium-bound enzymes, application of the whole broth of *T. reesei* could not only lead to cost reduction but also to improved saccharification and enhanced ethanol yields [27–30]. These suggest that on-site enzyme production with *T. reesei* could be a possible alternative to purchasing cellulases for a bioethanol plant using spruce as raw material.

In the present study, on-site cellulase production in a full-scale bioethanol plant was modelled together with the whole ethanol production process, and the economic impact of the enzyme fermentation step on the ethanol production cost was assessed. Cellulases were assumed to be produced using a mutant of *T. reesei*, employing the whole crude fermentation broth of the fungus in the ethanol production step. The effect of varying the carbon source of enzyme fermentation, at constant protein and mycelium yields, was investigated through the whole process. Different mixtures of pretreated liquid fraction, slurry, and molasses were evaluated as feed for enzyme production.

## 2. Materials and Methods

### 2.1. Raw Material

The dry spruce chips contain 37.9% glucan, 9.9% mannan, 1.8% galactan, 4.3% xylan, 1.3% arabinan, and 28.0% lignin. These values were derived from compositional analyses performed in EU-project NILE (contract no. 019882) according to the standardized method of National Renewable Energy Laboratory (NREL, Golden, CO) [31]. The remaining part is made up of acetyl groups, extractives, and other compounds which were estimated from a previous study [6]. The dry matter (DM) content was assumed to be 50%. Theoretically, 356 L of ethanol could be produced from the hexose sugars per dry tonne of raw material.

### 2.2. Overall Process Description

The proposed ethanol plant is assumed to be located in Sweden and process 200 000 dry tonne spruce chips annually. It is run by 28 employees, and is assumed to be in operation for 8000 hours per year.

**Figure 1:**
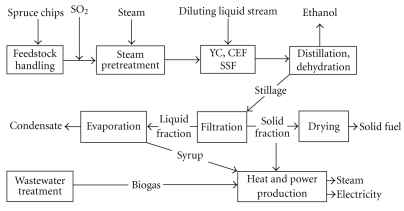
Overall process scheme for the proposed ethanol plant. In the reference case, there was no enzyme production; the enzymes were purchased. CEF: cellulase enzyme fermentation, YC: yeast cultivation and SSF: simultaneous saccharification and fermentation.

The process scheme is shown in Figure 1. Each step, except cellulase enzyme fermentation (CEF), has been described in detail elsewhere [6] and will only be discussed here briefly, focusing mainly on the minor modifications. Live steam was assumed to be available at 20 and 4 bar, and secondary steam is used to replace live steam whenever possible.

### 2.3. Reference Case

The conversion of carbohydrates is carried out in steam pretreatment and in simultaneous saccharification and fermentation (SSF) (Figure 1). Process data for steam pretreatment (210°C, 2.5% SO_2_) and SSF were based on results recently obtained from experimental work performed at the Department of Chemical Engineering, Lund University, Sweden.

Water needed to adjust the dry matter in the SSF step to 10% water-insoluble solids (WIS) is added before pressing the pretreated slurry. The diluting stream consists of fresh water and part of the evaporation condensate. It also contains ammonia to neutralize the slurry. The pressed liquid supplemented with molasses containing 50% sucrose is used in yeast cultivation (YC) without adding extra fresh water, hence the inhibitor concentrations in YC and SSF are approximately the same. Yeast seed train consisting of three stages provides SSF with 7.5% inoculum. Only the first and second stages are designed to be sterile, that is, those vessels are pressure-rated for steam sterilization. In SSF, the concentration of ordinary baker's yeast and the enzyme dosage are 3 g DM/L and 10 FPU (filter paper unit)/g WIS, respectively. The SSF takes place in twelve agitated nonsterile fermentors with a total volume of 920 m^3^ each. An SSF cycle including filling, fermentation, draining, and cleaning lasts for 60 hours. The number of the YC fermentors was calculated from the cycle time, which was assumed to be 15 hours for all YC stages.

According to the model calculations the ethanol content of the SSF broth is 3.8 wt-% which corresponds to a concentration of 40.4 g/L in the liquid phase. Distillation and molecular sieve adsorption are used to produce pure (99.8 wt-%) ethanol. The distillation step consists of two stripper columns and a rectifier, which are heat integrated by operating at different pressures. The remaining water in the overhead vapour leaving the rectifier is removed in the dehydration columns that are regenerated with pure ethanol vapour. The regenerate is returned to the rectifier.

The stillage of the stripper columns is separated in a filter press resulting in a solid fraction with a WIS content of 40%. The liquid fraction of the stillage is concentrated to 60% DM in an evaporation system which contains five effects in a forward-feed arrangement, that is, only the first effect is heated by live steam; the subsequent ones utilize the vapour from the previous effect, operating at higher pressure. Boiling point elevation was accounted for [32], and overall heat transfer coefficients were estimated to vary between 500 and 2000 W/m^2°^C, depending on the temperature and concentration of the liquid. Based on the work of Olsson and Zacchi [33], it was assumed that by applying a stripper column after evaporation, recycling of part of the evaporation condensate to dilute the whole slurry was possible. The rest of the condensate is sent to the wastewater treatment facility, where together with the condensed flash streams mainly originating from the pretreatment, it is treated by anaerobic digestion followed by an aerobic step [6]. It was assumed that 50% of the chemical oxygen demand (COD) was converted with a yield of 0.35 m^3^ methane/kg COD consumed.

Steam and electricity are generated by burning the biogas, the concentrated liquid fraction and part of the solid fraction of the stillage. The generated steam is allowed to expand to 4 bar through the turbine system, however, part of the steam is withdrawn at 20 bar for pretreatment and drying. The heat from flue gas condensation could be utilized by integrating a district heating system with the heat and power producing facility, however, this was not included in the model. The excess solid residue, that is, the solid fraction not required for steam generation, is dried in a superheated steam dryer to 88% DM. The secondary steam generated by drying is utilized in the process.

### 2.4. Description of Enzyme Fermentation

Process data for CEF were obtained from the literature [34, 35], however, some key assumptions were also made. The applied *Trichoderma *strain was assumed to be able to produce cellulase enzymes in the presence of monosaccharides, that is, it was not catabolite-repressed (e.g., *T. reesei* RUT C30). The mycelium, soluble protein, and activity yields were 0.27 g, 0.26 g, and 185 FPU per g carbohydrate in anhydro equivalent [35], respectively, which resulted in a specific activity of 710 FPU/g protein. After complete hydrolysis of polysaccharides, all the monosaccharides are consumed entirely in CEF, while other compounds are not involved in any reaction. Based on the work of Szengyel and Zacchi [36], it was assumed that inhibition due to compounds present in the pretreated material, such as furan derivatives and organic acids, did not occur.

The 5% inoculum is received from the second stage of a two-stage seed train. Both stages operate with 5% inoculum at a cycle time of 30 hours. The first stage receives inoculum from a stock culture, while the second is inoculated with the broth of the first. Concerning the composition, the seed stages are assumed to be run on the same feed as the production stage, where 120 hours cycle time is presumed. As this time is double of the cycle time of SSF, the number of vessels are 24 in the enzyme production stage. Considering the ratio of the cycle times of seed and production stages, the number of seed vessels is 6 in both stages. In all scenarios these numbers were kept constant, hence the total vessel volume varied in a range of 37–121 m^3^ in the production stage. The fermentors of the seed train are pressure-rated and can be sterilized at 120°C, however, it was assumed at the production stage that sterilization was not necessary. Cleaning-in-place is sufficient, since the evaporation condensate and the pretreated material were considered to be sterile and the fresh water added before pressing the pretreated slurry is sterile-filtered beforehand. Furthermore, the nutrients (soy-meal 0.5%, (NH_4_)_2_SO_4_ 0.15%, KH_2_PO_4_ 0.07%, and FeSO_4_ *·*7H_2_O 0.001%) and the molasses were assumed not to cause any contamination, so the seed vessels can be sterilized empty. At all stages, 30°C and pH 5 are kept. The feed is cooled down in a heat exchanger and the heat released during fermentation is removed by cooling water that circulates in jackets at the first stage and in coils at later stages. The cooling jacket is favourable in terms of cleaning, however, it is not sufficient at larger volume. The pH is controlled using ammonia. Aeration of 0.5 VVM was assumed to ensure sufficient agitation when the solid content was less then 1% WIS. The whole broth containing mycelia and enzymes is added to SSF. This can be done, since SSF is carried out at 37°C, and above 35°C the growth of mycelia is completely inhibited [35].

### 2.5. Enzyme Fermentation Configurations

**Figure 2:**
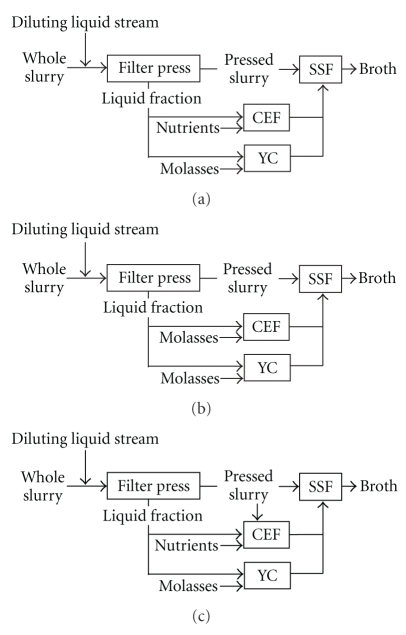
Layout of cellulase enzyme fermentation (CEF), yeast cultivation (YC) and simultaneous saccharification and fermentation (SSF) at scenarios A to C (a–c, respectively).

Three configurations, denoted with A-C, were investigated in the model of enzyme fermentation (Figure 2). They differed in the carbon source. In scenario A, part of the liquid fraction of the diluted slurry was used, while in scenario B, the liquid fraction was supplemented with molasses to increase the sugar content. In scenario C, a mixture of the liquid fraction and the pressed slurry was prepared, and used as feed for CEF. Scenario C was divided into three subcases, C1–3 depending on the WIS content of the mixture, that is, 1%, 2%, and 3%. For scenarios A and B, sensitivity analysis was performed, denoted with +. The specific activity of the soluble proteins was enhanced 1.5-fold, resulting in an increase of 50% in the productivity in terms of enzyme activity, while protein and mycelium yields remained the same.

The liquid fraction also contained water-insoluble particles, as a WIS retention of 99% was assumed in the filtration of the slurry. In the CEF feed the total carbohydrate content expressed in monomer equivalent (ME) and WIS concentration, in parentheses, were the following: A: 4.6% (0.5%), A+: 4.7% (0.7%), B: 10% (0.8%), B+: 10% (0.9%), C1: 4.9% (1%), C2: 5.5% (2%), and C3: 6.0% (3%). In cases C1–3, aeration was assumed to be insufficient for ensuring homogeneity, therefore agitators were built in, and power-to-broth value was set to 40 W/m^3^. In scenario B, molasses served as a complex nutrient source, hence nutrient supplementation was omitted.

### 2.6. Analysis Methods

Mass and energy balances were solved using the commercial flowsheeting program Aspen Plus 2006.5 (Aspen Technology, Inc., Cambridge, MA). Physical property data for biomass components such as polysaccharides and lignin were derived from the NREL database [37]. Fixed capital investment (FCI) costs were estimated either with Aspen Icarus Process Evaluator 2006.5 (Aspen Technology, Inc.) or from vendor quotation. The construction material was assumed to be 304 stainless steel for all process vessels. To obtain the annual FCI, an annuity factor of 0.110 was used, corresponding to a depreciation period of 15 years and an interest rate of 7%. Working capital investment (WCI) was calculated according to the recommendations in literature [38]. Annual WCI is the product of WCI and interest rate.

All costs are presented in Swedish kronor (SEK, 1 US$ *≈* 7.3 SEK, 1 *€* *≈* 10.5 SEK). In the reference case the purchase price of enzyme was assumed to be 28.5 SEK per million FPU. Cost of nutrients (in SEK/kg) applied in CEF were the following: soy-meal 1.5, (NH_4_)_2_SO_4_ 0.9, KH_2_PO_4_ 1.0, FeSO_4_ *·*7H_2_O 1.0. Cost of raw material, chemicals, utilities, labour, insurance, maintenance, and revenues of ethanol, coproducts, and electricity are reported in recent studies [6, 13]. Minimum ethanol selling price (MESP) refers to the ethanol price at break-even point, that is, at this price, annual cost and income are equal.

## 3. Results and Discussion

### 3.1. Process Design: The Effects of On-Site Enzyme Fermentation

After steam pretreatment, a slurry with a WIS content of 27% was obtained, which was much higher than that in experimental work. This was due to lower heat losses applied in the model (10% of the adiabatic heat demand). The whole slurry was diluted to a WIS content of 10.1%-10.2%, depending on the scenario, before pressing.

Components fed into CEF and SSF on a yearly basis, other features of CEF and overall ethanol yield are shown in Table 1. On-site enzyme production has the advantage that pentoses, not fermentable by ordinary baker's yeast, can also be utilized. Nevertheless, in regard to hexoses, CEF competes with ethanol fermentation, since the more sugar is used for CEF, the less sugar is available for ethanol production. The highest mass flow of C_5_ sugars (pentosans and pentoses in ME) into CEF was obtained in scenario A, which corresponded to 13% of C_5_ sugars recovered after pretreatment, hence even at the best utilization, 87% was burnt or dried. Regarding the composition of sugars in the CEF feed, scenarios A and A+ gave the highest C_5_ proportion (21%). This would increase considerably, if the raw material of the ethanol plant consisted of more pentosans, for example, if agricultural byproducts were used instead of softwood. In scenarios A, A+, and C1–3, the C_5_ ratio decreased monotonously with increased WIS content in the feed, since the solid fraction contained relatively less C_5_ sugars compared to the sugar composition of liquid fraction. In the cases supplemented with molasses (B and B+), the C_5_ ratio was only 9%, due to the high ratio of sugars of molasses (56%-57%). The productivity of CEF varied in a range of 70–230 FPU/(L*·*h) (Table 1). As the fermentation time was maintained at 108 hours in each scenario, the productivity only depended on the carbohydrate concentration and activity yield.

Although CEF decreased the C_6_ flow (hexosans and hexoses) in SSF feed, the majority of C_6_ sugars were fermented to ethanol (Table 1). Even in the worst scenario (C3), the decrement of C_6_ sugars (in ME) was only 5850 tonne/year, which corresponded to 6% of the C_6_ sugars (in ME) fed to SSF in the reference case. The yeast amount required by SSF slightly decreased with increasing WIS contents in CEF (Table 1), since by feeding more WIS into CEF, the WIS flow fed to SSF decreased, which resulted in smaller total fermentation volume in SSF at constant WIS concentration. Excluding the cases of 1.5-fold specific activity, the produced enzyme protein varied little (Table 1). The produced amount depended on the WIS flow fed into SSF, hence the highest amount was obtained for scenario B due to the lowest WIS consumption in CEF. Mycelium had the same trend as enzyme protein, since their ratio was fixed by their yields.

The ethanol concentration in the SSF broth varied between 3.6 and 3.7 wt-%, which corresponded to a range of 38.1 to 39.7 g/L in the liquid phase. They were slightly lower than in the reference case, due to drop of the contents of hexoses and hexosans in the liquid and solid phases, respectively. The overall ethanol yield of the process in CEF cases also decreased compared to the reference (Table 1). The decrements were lowest in scenarios B and B+ owing to the large proportion of sugars coming from molasses. The yields in scenarios A and C1–3 were equal, which implied that the model was indifferent to the WIS content of CEF in the range investigated.

### 3.2. Economics: Specific Enzyme Cost, Minimum Ethanol Selling Price and Annual Cash Flows

**Figure 3:**
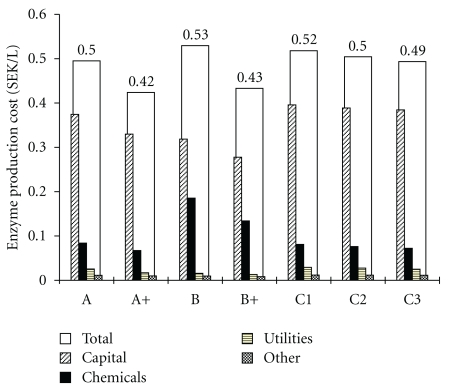
Cost contributors of enzyme production in SEK/L ethanol. Molasses was added to chemicals. Other refers to maintenance and insurance. No extra labour was accounted for the enzyme fermentation area. Carbon source A: pretreated liquid fraction, B: pretreated liquid fraction and molasses and C: pretreated liquid fraction and pressed pretreated slurry with a total WIS content of 1%, 2% and 3%; +: 1.5-fold specific activity.

Cost elements of enzyme production expressed in SEK/L ethanol are shown in Figure 3. Capital and chemical costs were found to be the main contributors in each scenario. Utilities refer to the electricity consumption of the compressor and the agitators (scenarios C1–3) and to the demand for cooling water. The cost for steam used for sterilization of the empty seed vessels was assumed to be negligible. The lowest capital costs were obtained in scenarios B and B+, due to high carbohydrate concentration in CEF, which resulted in small fermentor volume. However, the cost of chemicals was the highest in these scenarios, owing to the extra cost of molasses. In cases C1–3, increasing the WIS concentration in CEF resulted in a reduced fermentation volume, and in consequence, lower capital and chemical costs.

**Figure 4:**
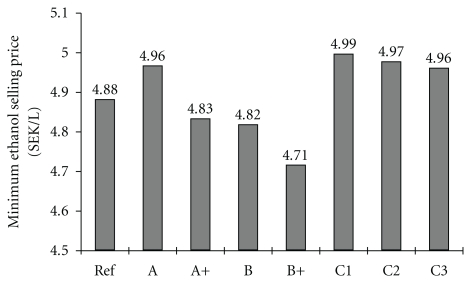
Minimum ethanol selling price. Carbon source A: pretreated liquid fraction, B: pretreated liquid fraction and molasses and C: pretreated liquid fraction and pressed pretreated slurry with a total WIS content of 1%, 2% and 3%; +: 1.5-fold specific activity; Ref: reference case with purchased enzyme preparation.

Comparing the base scenarios (A, B, and C1–3), case B proved to have the highest total cost of enzyme production, due to the additional cost of molasses. On the other hand, in regard to MESP, case B was the most favourable, furthermore, this was the only base scenario with on-site enzyme production in which the MESP was lower than that in the reference case, with purchased enzymes (Figure 4). In spite of the extra expenses, molasses could improve the process economics considerably, since CEF supplemented with molasses reduced the overall ethanol yield, the most important parameter in the production cost of ethanol [39], to a smaller extent.The 1.5-fold activity yield resulted in a decrease of 16 and 19% in total enzyme production cost compared to the base scenarios A and B, respectively, which corresponded to a decrement of 2.6 and 2.3% in MESP.

Annual cash flows are presented in Table 2, calculated for a selling price of ethanol of 5.5 SEK/L. The CEF increased the capital costs significantly (10%–14%) compared to the reference case. The second largest cost contributor after the capital cost was the raw material cost, which did not change due to constant annual capacity. Also these costs have been proved to be the main contributors to the production cost of lignocellulosic ethanol in previous, similar studies [3, 6, 7, 13]. Chemical expenses increased by 12%–34% compared to the reference case. The utility costs were the lowest among the cost elements in each scenario, since only process and cooling water had to be purchased, as steam and electricity were generated on-site.

Ethanol, the main product, gave 83%-84% of the annual revenues. Co-products refer to solid fuel, that is, the dried excess solid residue, and the carbon-dioxide produced in CEF, YC, and SSF, which was also assumed to be marketable. Solid fuel contributed to 97% of the co-products income in each scenario. While steam generation met the steam requirement of the process, produced electricity was consumed on-site only partially. The excess electricity varied between 35% and 41% of the amount produced, the lowest being in cases C1–3, due to the consumption of agitators, whereas the highest being in the reference case. The highest profit was achieved in scenarios B and B+, where the MESPs were the lowest. Table 2 clearly shows the importance of co-product and electricity revenues, since the income of ethanol does not exceed the expenses.

## 4. Conclusions

By means of the developed model of on-site enzyme production, embedded in a softwood-based ethanol process, various streams were studied as carbon sources in enzyme fermentation at constant soluble protein and mycelium yields. The majority of sugars consumed in enzyme fermentation were C_6_, especially when molasses was present. The overall ethanol yields were lower in those scenarios, where enzyme fermentation was included, than in the reference case, where a purchased cellulase preparation was applied. This was due to the fact that enzyme production decreased the amount of carbohydrates available for the yeast to produce ethanol. When molasses was used as additional carbon source the minimum ethanol selling price was the lowest among the scenarios, resulting in the highest annual profit, although the specific enzyme production cost was found to be the highest. 

On-site enzyme fermentation contributed to 9%–11% of the ethanol production cost. The feasibility of including enzyme production in the lignocellulosic ethanol process highly depends on the full-scale price of commercial cellulase enzyme preparation, which is still very uncertain. At the premises of the study, some scenarios proved to be more feasible than that with purchased enzymes, which implies that on-site enzyme production can be an alternative. To achieve further improvement in the economics of a process integrating cellulase fermentation, the enzyme demand of SSF has to be decreased, whereas the activity yield and productivity, the two most important parameters of enzyme fermentation in terms of cost reduction, have to be increased.

Similarly to previous evaluations [6, 7], the present study demonstrated the importance of the overall ethanol yield and the co-product revenues in regard to the process economics. On-site enzyme production is the most feasible, when the least C_6_ sugars are consumed, hence it decreases the overall ethanol yield to a smallest extent. A plant using a raw material with higher C_5_ proportion, such as agricultural by-products, could become relatively more viable by integrating enzyme fermentation. However, further investigation is required to prove this statement.

## Figures and Tables

**Table 1:** Features of enzyme and ethanol productions. Both CEF and SSF are carried out in batch operation. Carbon source A: pretreated liquid fraction, B: pretreated liquid fraction and molasses, and C: pretreated liquid fraction and pressed pretreated slurry with a total WIS content of 1%, 2%, and 3%; +: 1.5-fold specific activity.

	**Reference**	**A**	**A+**	**B**	**B+**	**C1**	**C2**	**C3**
Components into CEF, tonne/year								
Hexosans	—	430	368	298	236	776	1374	1861
Pentosans	—	7	6	5	4	13	23	32
Hexoses	—	5133	3352	6242	4120	4812	4256	3805
Pentoses	—	1487	971	622	413	1394	1233	1102
WIS^a^	—	806	690	558	442	1453	2575	3486
C_5_ ratio in CEF^b^, %	—	21	21	9	9	20	18	16
WIS content in CEF, %	—	0.5	0.7	0.8	0.9	1.0	2.0	3.0
Productivity of CEF^c^, FPU/(L*·*h)	—	70	107	154	230	75	84	93

Components into SSF, tonne/year								
Hexosans	62980	62550	62612	62683	62745	62205	61606	61120
Hexoses	32634	27521	29304	30499	31228	27841	28397	28848
Yeast	3168	3160	3160	3160	3168	3144	3128	3112
Enzyme protein	1816^e^	1656	1104	1682	1121	1651	1642	1635
Mycelium	—	1727	1152	1754	1170	1722	1713	1705
WIS^a^, ktonne/year	117.98	117.55	117.61	117.68	117.74	117.19	116.59	116.09

Overall EtOH yield^d^, L/dry tonne	270	254	260	263	266	254	254	254

^a^Includes hexosans and pentosans.

^b^(pentosans  in  ME + pentoses)/total sugar in ME.

^c^Corresponds to an enzyme fermentation time of 108 h.

^d^Includes yeast and enzyme productions and ethanol losses in the process.

^e^The purchased enzyme preparation was assumed to contain 10% protein.

**Table 2:** Annual costs, revenues, and profit of the proposed ethanol plant in MSEK. Carbon source A: pretreated liquid fraction, B: pretreated liquid fraction and molasses, and C: pretreated liquid fraction and pressed pretreated slurry with a total WIS content of 1%, 2%, and 3%; +: 1.5-fold specific activity.

	**Reference**	**A**	**A+**	**B**	**B+**	**C1**	**C2**	**C3**
Annual cost (MSEK)								
Raw material	99.5	99.5	99.5	99.5	99.5	99.5	99.5	99.5
Capital	135.5	152.7	151.6	151.0	149.1	154.2	153.8	153.5
Chemicals	28.3	32.4	31.7	38.0	35.4	32.2	31.9	31.6
Enzymes	33.6	—	—	—	—	—	—	—
Utilities	2.3	2.5	2.4	2.5	2.4	2.5	2.4	2.4
Other	20.8	21.3	21.2	21.2	21.2	21.3	21.3	21.3

Total	320.0	308.3	306.4	312.2	307.6	309.7	309.0	308.3

Annual income (MSEK)								
Ethanol^a^	297.4	279.8	285.7	289.7	292.2	279.8	279.7	279.7
Co-products	45.1	45.9	45.3	48.5	46.8	46.1	46.5	46.8
Electricity	11.1	9.8	10.2	10.0	10.3	9.5	9.4	9.4

Total	353.5	335.6	341.2	348.2	349.3	335.4	335.7	335.8

Annual profit (MSEK)	33.6	27.2	34.8	36.0	41.7	25.7	26.7	27.5

^a^Ethanol price was assumed to be 5.5 SEK/L.

## Abbreviations

CEF: Cellulase enzyme production
COD: Chemical oxygen demand
DM: Dry matter
FCI: Fixed capital investment
FPU: Filter paper unit
ME: Monomer equivalent
MESP: Minimum ethanol selling price
NREL: National renewable energy laboratory
SEK: Swedish kronor
SSF: Simultaneous saccharification and fermentation
WCI: Working capital investment
WIS: Water-insoluble solids
YC: Yeast cultivation
